# Blood Clot Formation Does Not Affect Metastasis Formation or Tumor Growth in a Murine Model of Breast Cancer

**DOI:** 10.1371/journal.pone.0094922

**Published:** 2014-04-16

**Authors:** Stephanie Rossnagl, Anja von Au, Matthaeus Vasel, arco G. Cecchini, Inaam A. Nakchbandi

**Affiliations:** 1 Max-Planck Institute of Biochemistry, Martinsried, Germany; 2 Institute of Immunology, University of Heidelberg, Heidelberg, Germany; 3 Department of Urology, University of Bern, Bern, Switzerland; University of Alabama at Birmingham, United States of America

## Abstract

Cancer is associated with increased fracture risk, due either to metastasis or associated osteoporosis. After a fracture, blood clots form. Because proteins of the coagulation cascade and activated platelets promote cancer development, a fracture in patients with cancer often raises the question whether it is a pathologic fracture or whether the fracture itself might promote the formation of metastatic lesions. We therefore examined whether blood clot formation results in increased metastasis in a murine model of experimental breast cancer metastasis.

For this purpose, a clot was surgically induced in the bone marrow of the left tibia of immundeficient mice. Either one minute prior to or five minutes after clot induction, human cancer cells were introduced in the circulation by intracardiac injection. The number of cancer cells that homed to the intervention site was determined by quantitative real-time PCR and flow cytometry. Metastasis formation and longitudinal growth were evaluated by bioluminescence imaging.

The number of cancer cells that homed to the intervention site after 24 hours was similar to the number of cells in the opposite tibia that did not undergo clot induction. This effect was confirmed using two more cancer cell lines. Furthermore, no difference in the number of macroscopic lesions or their growth could be detected. In the control group 72% developed a lesion in the left tibia. In the experimental groups with clot formation 79% and 65% developed lesions in the left tibia (p = ns when comparing each experimental group with the controls). Survival was similar too.

In summary, the growth factors accumulating in a clot/hematoma are neither enough to promote cancer cell homing nor support growth in an experimental model of breast cancer bone metastasis. This suggests that blood clot formation, as occurs in traumatic fractures, surgical interventions, and bruises, does not increase the risk of metastasis formation.

## Introduction

Breast and prostate cancer represent the most common solid tumors in adults associated with bone metastasis [1]. These metastases originate from circulating cancer cells that hijack the hematopoietic stem cell niches in the bone marrow taking advantage of its unique richness in cytokines [2]–[4]. The growth of a metastatic lesion in the bone often increases the risk of a pathologic fracture [5], [6]. These fractures are mostly predictable [7] and largely contribute to a worsened quality of life in patients with metastatic bone disease [5]. While most fractures occur in the presence of a metastatic lesion, cancer is often associated with osteoporosis and hence an increase in fracture risk [8]. Occasionally, a fracture site is later found to contain metastatic disease. Therefore the question occasionally arises as to whether the occurrence of a fracture in a patient with cancer is a reflection of the presence of a metastatic lesion at the fracture site or whether the pathologic processes that take place in the event of a fracture increase the risk of establishment of tumor cells at the site of the fracture.

One of the first events that take place after a fracture is the development of a hematoma, in which the coagulation cascade is activated. Blood clots include a number of proteins that have been shown to directly affect tumor development. Thrombin, a terminal clotting protein, supports cancer implantation and growth [9]. Factor XIII stabilizes thrombi and supports metastasis formation by interfering with natural-killer mediated cancer cell removal [10]. Fibrinogen, another molecule involved in the clotting cascade was shown to support cancer cell adhesion and survival [11]. Other participants in the coagulation cascade such as tissue factor have been associated with metastatic disease in correlative studies and a causative role is presumed albeit not proven [12], [13]. Furthermore, the platelets themselves produce SDF-1 (stromal-cell derived factor-1), which can act as a chemotactic agent for cancer cells [14]. Thus, molecules upregulated in the early stages of clot formation or in fracture hematomas and proteins concentrated there as a result of coagulation activation that support infiltration by inflammatory cells can also be involved in tumor development. Indeed, interfering with some of these events seems to negatively affect cancer development [15], [16].

Based on these and other studies one might be inclined to conclude that the formation of a blood clot as might occur in fractures is associated with the development of metastatic disease. We therefore aimed to test whether the development of a blood clot can be directly responsible for the formation of a metastatic lesion. This seems particularly relevant in view of observational studies suggesting that events associated with tooth extraction are enough to increase the rate of metastasis formation [17]. To achieve this aim, we used an experimental model, in which a blood clot is induced in the left tibia. Cancer cells selected to home to the bone marrow were then introduced in the circulation by means of intracardiac injection to ensure the presence of large numbers of circulating cancer cells at the time of clot formation [18]. Using this model we examined the homing of cancer cells to the blood clot in the bone marrow in the left tibia in comparison to the opposite side that did not undergo clot induction. We also compared the development of metastatic lesions in these mice to control mice that did not undergo clot induction. We found neither an increase in the number of cancer cells localized to the clot nor an increase in the number of metastatic lesions developing in the injured left tibia. This suggests that the formation of clots/hematomas, albeit rich in growth factors does not provide optimal conditions for cancer growth. Thus there is currently no evidence to support fear of increased metastasis formation after clot formation as might occur in fractures and surgical interventions.

## Methods

### Mice

CD1 nu/nu animals were obtained from Charles River Laboratories (Kissleg, Germany). These mice carry a *foxn* mutation that results in their inability to produce functional T cells and therefore these animals are suited for a xenotransplant model. In addition, hair follicle development is impaired, and hence these mice are nude, allowing for bioluminescence imaging without shaving.

The studies in mice were approved by the animal protection committee of the University of Heidelberg and Regierungspräsidium Karlsruhe #G48/08, #G120/11, #G73/13 and #G136/13, thus, all animal work was conducted according to relevant national and international guidelines. All surgery was performed under anesthesia, and all efforts were made to minimize suffering.

### Cancer cells

MDA-MB-231B/luc^+^ or PC-3M-Pro4/luc^+^ were cultured in DMEM/10%FCS with 800 and 500 µg/ml geneticine respectively [18]. Huh-7 hepatoma cells were obtained from Cell Bank, Japan: JCRB0403) and cultured in DMEM/10% FCS. Cells were counted using an automated cell counter (CASY-TT, Innovatis).

### Intracardiac injection of cancer cells

For intracardiac injection of cancer cells, mice were anesthetized (Ketamine 120 mg/kg/xylazine 16 mg/kg). A cancer cell suspension (10^5^/100 µl PBS) (MDA-MB-231 selected to home to the bone marrow and establish bone metastases), PC3 or Huh-7 was injected into the left heart ventricle [18]. Tumor growth was evaluated weekly starting 3 weeks after intracardiac injection by bioluminescence reporter imaging.

### Induction of blood clot

Intratibial bone marrow flushing was performed as described [18], but without injecting cancer cells intratibially. Briefly, mice were anesthetized [18], [19], skin and muscle were cut and pushed aside from over the left tibia, two holes, 3–4 mm apart with a diameter of ∼0.35 mm each, were drilled with a dental drill (Bredent) through bone cortex. Bone marrow was flushed out with 0.5 ml phosphate buffered saline injected in the upper hole, which was then sealed with surgical bone wax (Ethicon; Johnson and Johnson) together with the lower hole. Lastly, the cutaneous wound was sutured. In the homing experiment, the opposite side was not operated upon to maximize the difference, but in the growth experiment the control group underwent sham operations without drilling or flushing.

### Bioluminescence imaging

For bioluminescent reporter imaging that allows following bone metastasis growth longitudinally, mice were anesthetized with isofluran and injected with d-luciferin (150 mg/kg body weight)(Synchem). Exactly 5 minutes after injection, photon signal was detected using an “IVIS-100” imaging system, and evaluated using the analysis software “Living Image” (version 2.50).

### X-ray analysis

Lytic lesions were detected by radiography using a Faxitron. Lytic lesions on x-rays were analyzed using “Image J” (Wayne Rasband, NIH).

### Staining protocols, histomorphometry and determination of clot area

Bones were fixed in 3.7% neutral-buffered formalin (NBF), embedded in polymethylmethacrylate, sectioned and stained per Masson Goldner with hematoxilin (Gill II, Carl Roth, Karlsruhe, Germany), acid fuchsin-ponceau xylidine, and phosphomolybdic acid-orange G and light green [20]. For dynamic histomorphometry, calcein was administered twice, once immediately after clot induction and then 48 hours later intraperitoneally at 30 mg/kg (Sigma-Aldrich, Munich, Germany), and mice euthanized 24 hours later. Primary cancellous bone was defined as the 120 µm band below the growth plate. Cancellous bone was defined as the remaining trabecular area that extends down 2 mm [21]. The same sections were used for dynamic and static histomorphometry, and data obtained from evaluation of the cancellous bone area defined above are presented. The ASBMR nomenclature was used [22]. The following measurements are mentioned: osteoid surface (OS), bone surface (BS), osteoblast number (Ob.N), bone formation rate (BFR = MS*MAR/BS, mm2/mm/yr.), number of osteoclasts (Oc.N), and erosion surface (ES). ImageJ was used (Wayne Rasband, NIH). Staining for thrombin was performed on plastic sections using a polyclonal antibody directed against thrombin (Abcam 92621) for one hour. The secondary antibody used was a goat anti-rabbit antibody labeled with Alexa 647 (Abcam 150079). Blood clot size was determined after initial screening of the sections to determine the section with the largest clot size. The selected sections were stained per Masson-Goldner. The apparent hematoma area after 5 min, 24 h and 48 h of clot induction was analyzed using ImageJ (Wayne Rasband, NIH). To perform enzymatic stains 5 µm cryosections of 3.7% neutral-buffered-formalin-fixed bones were performed using adhesive film (SECTION-LAB Co. Ltd.) as described [23]. Until further use the sections were stored at −80°C. TRAcP (tartrate-resistant acid phosphatase) to detect osteoclasts was stained as described [24]. Briefly: the slides were placed in dH2O and the following solution was prepared: 16 mg Naphthol ASTR phosphate (Sigma) was dissolved in 1 ml dimethylformamide. The Naphthol ASTR phosphate was added to 10 ml 0.1 M acetate buffer with Pararosaniline and the pH adjusted to 5.0. Finally 3 drops of Manganese sulfate were added. The wet slides were incubated 4 mins with the staining solution and after gently rinsed with dH2O and mounted with Mowiol. Alkaline phosphatase staining to detect osteoblasts was performed as described [25]. Briefly, slides were placed in Tris buffer. And the following staining solution prepared: 40 mg Naphthol ASBI phosphate (Sigma) was dissolved in 2 mL of dimethylformamide (Merck). 40 mg of Fast Blue RR salt (Sigma) was dissolved in 2 mL of dimethylformamide. To prepare the final staining solution, 2 mL of naphthol ASBI solution was combined with 2 mL of Fast Blue RR salt solution. This was then added to 35 mL of Tris buffer (pH 9.4, Roth). The solution was filtered before use and was prepared fresh as the substrate deteriorates over time. The slides were incubated for 2 min in the staining solution. After incubation in stain, slides were rinsed in dH2O and mounted with Mowiol. Alkaline phosphatase–rich structures are stained a dark blue color. The number of TRAcP osteoclasts was counted and the surface of alkaline phosphatase stain was measured and adjusted to total bone surface as described [26]. Sections were photographed using a Keyence microscope and processed using ImageJ. Quantification was performed in at least three mice per group or more as noted in the figure legends.

### DNA analysis

For evaluating the homing of circulating tumor cells mice were sacrificed 1 h, 4 h, 24 h and 48 h after intracardiac injection and bones and bone marrow taken for further analysis.

Genomic DNA was isolated from bone marrow using DNeasy Blood and Tissue kit (Qiagen). Quantitative real-time polymerase chain reaction (qPCR) was performed using a light cycler 2.0 Instrument (Roche) using the following primers and probes that detect resistance towards Geneticin in the construct introduced in the MDA and PC3 cell lines to allow bioluminescence imaging in a method similar to the use of the alu sequence described by other groups [27]. These primers however overcome the problem with contamination by human DNA and are as follows: forward 5′-3′: ACTGTTCGCCAGGCTCAAGGC, reverse 5′-3′ GCGAATCGGGAGCGGCGAT and probe #31. Huh-7 cells were detected using the following primers and probe: forward: 5′CAT GGT GAA ACC CCG TCT CTA 3′; reverse: 5′GCC TCA GCC TCC CGA GTA G 3′; probe: 5′ ATT AGC CGG GCG TGG TGG CG 3′. Results were normalized to mouse bone marrow cells using probe #64 and primers for β-actin (Universal probe library, Roche). An external standard curve using known numbers of human and mouse specific cells was created for Geneticin (for MDA and PC3), alu (for Huh-7) and β-actin (for murine bone marrow). Performing qPCR on bone and bone marrow DNA and comparing the results with a standard curve for tumor cells in mouse marrow we were able to detect as few as 0.2–0.5 human cancer cells/10^6^ murine bone marrow cells.

### Flow cytometry

Bone marrow was flushed from the upper third of the tibiae with 100 µl PBS/tibia, red cells lysed, and cancer cells stained with an APC-conjugated antibody directed against human CD49e, which is the integrin α5 subunit. This antibody does not bind to murine cells and detects 96% of MDA cancer cells (Clone NKI-SAM-1, Biolegend). The antibody was used at a final concentration of 0.25 µg/ml. Flow cytometry was performed using LSR-2 (BD-Biosciences), and at least 3 million cells were counted per sample.

### Statistical analyses

Analyses were performed using SPSS (V14.0). Comparisons between two groups were performed using Student's t-test or Wilcoxon paired test as appropriate. In the analysis for homing of cancer cells after different time periods from clot induction a one way ANOVA was first performed. Analysis of occurrence of tibial lesions was performed using Fisher's exact test. Calculations for sample size ahead of experiments to detect a difference with a power of 0.80 as well as post-hoc power analysis were performed using the PS program available online (http://biostat.mc.vanderbilt.edu/wiki/Main/PowerSampleSize). A test was defined as significant if p<0.05. Results are presented as mean±standard-error-of-the-mean (M±SEM).

## Results

### Intratibial hematoma and clot formation

**Figure 1:**
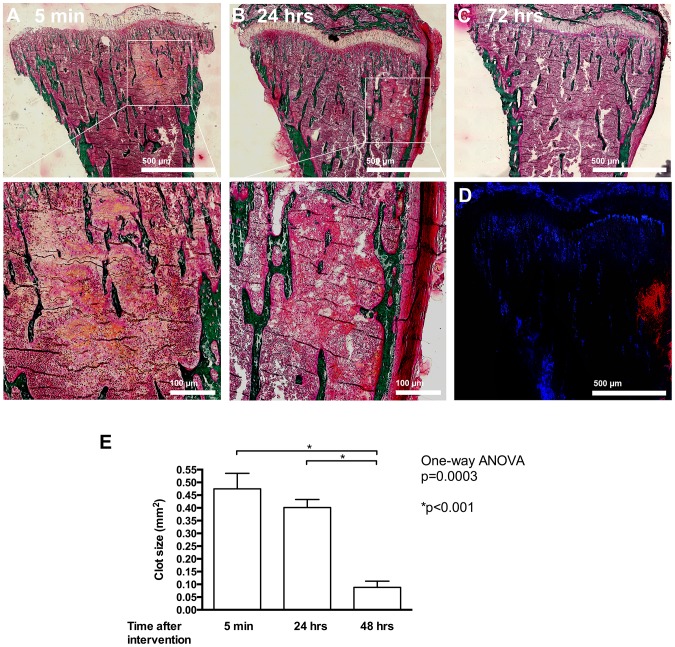
Induction of hematoma and clot formation in the bone marrow. (A) Induction of bleeding results in clot formation within 5 minutes as evidenced by Masson-Goldner staining. *Bar represents 500* µ*m*. The enlarged inset below shows the blood clot. *Bar represents 100* µ*m*. (B) The clot is still present, albeit partially reorganized at 24 hours after bleeding induction. *Bar represents 500* µ*m*. Enlarged inset is shown below. *Bar represents 100* µ*m*. (C) After 72 hours the clot resolved. (D) Thrombin staining of the bone shown in B confirms the presence of thrombin (in red) in the clot area. (E) Quantification of the change in hematoma size at different time points. After induction of bleeding in the bone marrow, mice were euthanized at the times mentioned, and tibiae examined. n = 3–4 mice/time point.

In order to determine whether the model we contemplated using indeed resulted in the development of a blood clot within the bone marrow, we performed the procedure of intratibial bone marrow flushing in mice and examined the tibiae 5 and 60 minutes, as well as 24, 48 and 72 hours after the end of the procedure. Sections within the tibia showed the formation of a clot already within 5 minutes after end of the procedure as seen with Masson Goldner staining (figure 1 A and inset below). Smaller clots were still detectable after 24 hours (figure 1B and inset), but by 72 hours the clot had almost completely resolved (figure 1C). The presence of the clot was further confirmed by thrombin staining (figure 1D). Quantification confirmed the decrease in size of the clot over time (figure 1E).

### Effect of blood clot induction on bone histomorphometry

**Figure 2:**
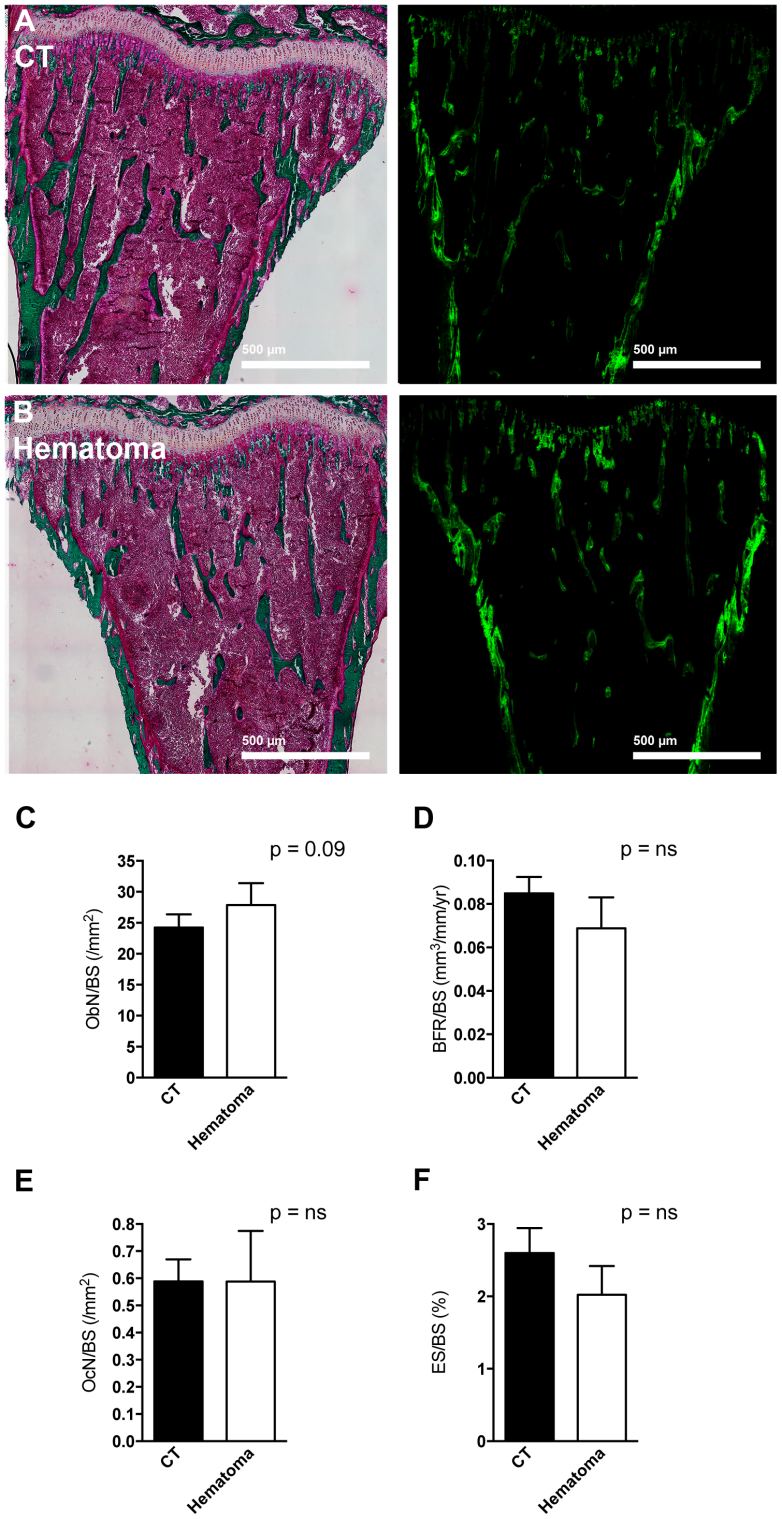
Bone histomorphometry after clot induction. (A–B) After three days from the time of induction of a blood clot Masson-Goldner staining (on the left) and calcein labeling (on the right) showed no difference between control (CT) in panel A and hematoma induction in panel B in the same mouse. Neither osteoblast number (C), nor bone formation rate (D), nor osteoclast number (E), nor erosion surface (F) were different between the two tibiae. CT represents the right tibia without clot induction and hematoma represents the left tibia in which bone marrow was flushed and a blood clot was induced. Tibiae were obtained three days after clot induction in the left tibia, embedded in polymethylmethacrylate and stained using Masson-Goldner. n = 4 mice.

**Figure 3:**
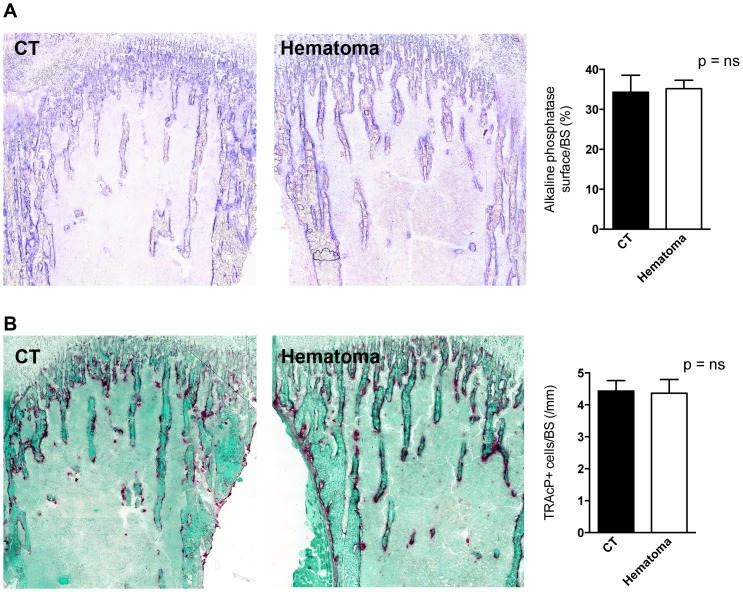
Enzymatic staining of bone sections. (A) Osteoblast surface as evidenced by evaluating the surface stained positive for alkaline phosphatase did not differ between CT and hematoma bones. A representative stained pair of tibiae is shown on the left and quantification is shown on the right. (B) The number of osteoclasts as evidenced by counting the cells that stained positive for tartrate-resistant acid phosphatase and correcting to bone surface was not affected by clot induction. A representative stained pair of tibiae is shown on the left and quantification is shown on the right. CT represents the right tibia without clot induction and hematoma represents the left tibia in which bone marrow was flushed and a blood clot was induced. Tibiae were obtained three days after clot induction in the left tibia, fixed in 3,7% PFA, cryo-sectioned using adhesive film and stained as outlined in the methods. n = 4 mice.

Cancer growth increases whenever there is an increase in bone turnover [28]. In order to determine whether flushing of the bone marrow and the development of a blood clot resulted in increased turnover we compared the flushed tibia with the opposite side tibia. The time point used was three days after the procedure to allow for cell changes to take place. As shown in figure 2, no obvious differences could be detected in bone sections. Both dynamic and static histomorphometric analyses were performed. Despite a trend to increased osteoblast numbers (p = 0.09), bone formation rate remained similar. Bone resorption was not affected as evidenced by similar osteoclast numbers and erosion surface on static histomorphometry. Using enzymatic staining we confirmed the absence of an effect on both osteoblasts (by measuring the surface of alkaline phosphatase staining) (figure 3A) and on osteoclasts (by counting the number of tartrate-resistant acid phosphatase-stained osteoclasts) (figure 3B).

Thus, induction of a blood clot does not affect bone turnover by the time the clot resolved.

### Intratibial hematoma formation is not associated with increased homing of cancer cells

**Figure 4:**
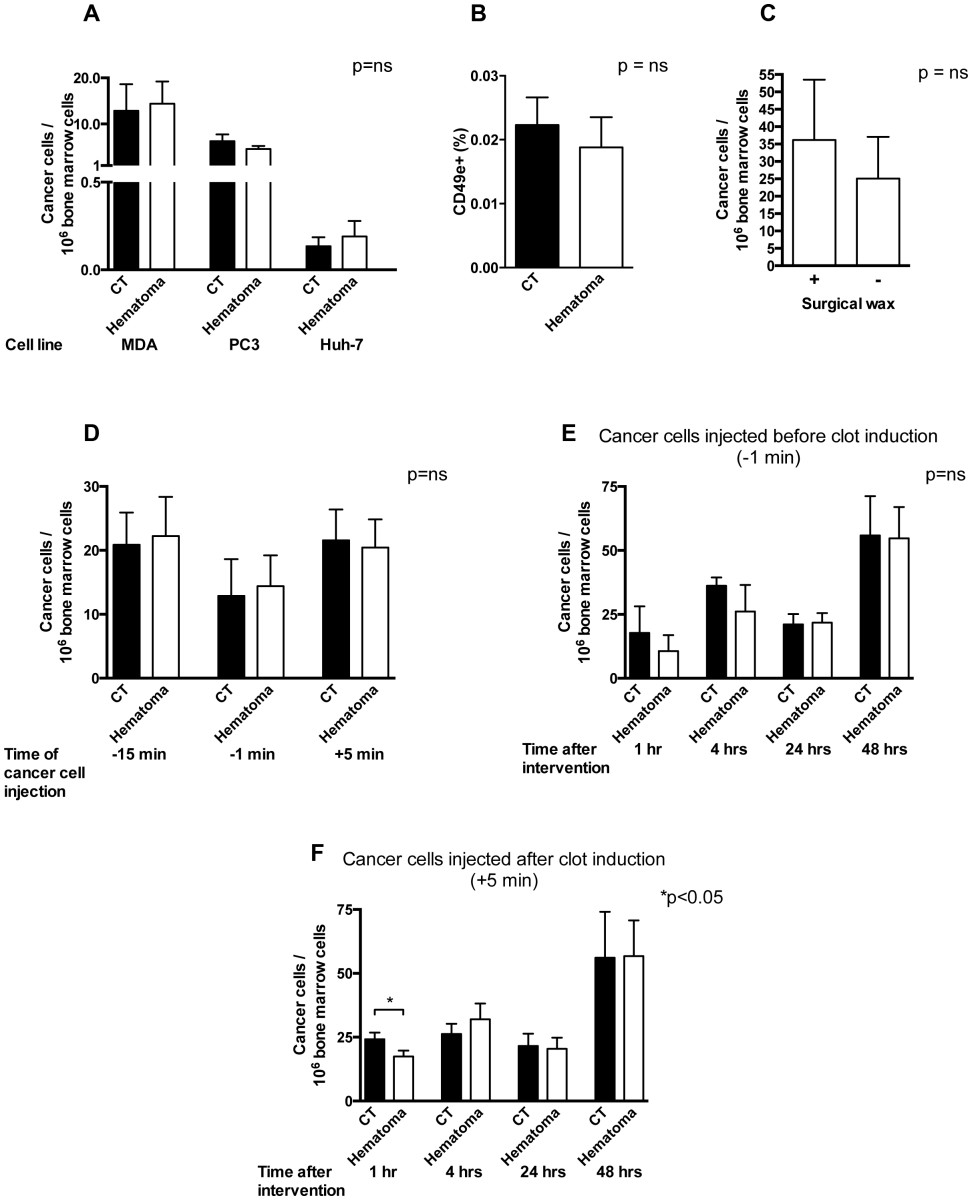
Infiltration of blood clots by cancer cells. (A) Induction of a blood clot in the left tibia does not result in an increase in the number of infiltrating cancer cells compared to the right control tibia (CT) in the same mouse when cancer cells are injected 1 minute before blood clot induction using three cell lines (Breast cancer selected to home to the bone marrow: MDA-MB-231B/luc+; prostate cancer able to form bone metastases: PC3/luc+; and hepatoma cells not reported to form bone metastases: Huh-7). Cancer cells were injected 1 minute before clot induction. 24 hours later the bone marrow was isolated from the upper third of both tibiae and the number of cancer cells was evaluated by quantitative PCR of a cancer cell specific sequence and corrected to the total number of murine cells in the sample. n = 4–5/group. (B) The number of MDA cancer cells evaluated by flow cytometry was similar between the CT and hematoma group. MDA cancer cells were injected 1 minute before clot induction. 24 hours later the bone marrow was isolated from the upper third of both tibiae, red cells lysed, stained with a labeled human-specific CD49e (integrin α5) antibody and at least 3 million bone marrow cells were counted. n = 10 mice. (C) The use of surgical wax in the tibia following clot induction does not affect homing of cancer cells. 1 minute before clot induction MDA cancer cells were injected. The hole performed in the tibia in order to induce the blood clot in the bone marrow was either closed with surgical wax or left until bleeding stopped spontaneously (3–5 minutes) before closing the wound. n = 4 pairs. (D) Injection of MDA cancer cells 15 minutes before, 1 minute before and 5 minutes after clot induction did not affect the number of cancer cells in the bone marrow detected after 24 hours. Samples were prepared as in A. p = ns for each time point. (E) Evaluation of cancer cell numbers when injected 1 minute before clot induction did not reveal a difference in the number of cancer cells detected in the bone marrow at different time points (1, 4, 24 and 48 hours after clot induction). p = ns and n = 4–5 per time point. (F) Evaluation of cancer cell numbers when injected 5 minutes after clot induction showed a significant decrease in the number of cancer cells detected in the bone marrow at 1 hour after clot induction (p<0.05) but not at later time points (4, 24 and 48 hours after clot induction) (p = ns). n = 4–8 per time point.

We then sought to examine whether the presence of a blood clot was associated with increased number of cancer cells arriving to the clot. We used three different cell lines, one that was selected to home to the bone marrow and form breast cancer metastases (MDA-MB-231B/luc+), one that forms prostate metastases in the bone (PC3/luc+) and a hepatoma cell line not reported to form bone metastases. Since these are human cell lines, they have to be used in immune-deficient mice lacking mature T-cells to avoid destruction of the human cells. Mice underwent intracardial injection of cancer cells followed one minute later by flushing of bone marrow in the left tibia. Twenty-four hours later the upper half of the left tibia (site of intratibial clot induction) was isolated and the number of tumor cells arriving at the site was evaluated using quantitative real time polymerase chain reaction (qPCR) to determine the number of cancer cells, whereby the frequency of a specific sequence found exclusively in the cancer cell line corrected to murine β-actin reflecting the number of murine bone marrow cells was used. As a control, the right tibia (site without clot induction) was evaluated. Injecting these cell lines intracardially one minute before clot induction was not associated with a change in cancer cells homing to the bone marrow (figure 4A). Using flow cytometry we were able to confirm the absence of a difference in the breast cancer cells (MDA-MB-231B/luc+) cells (figure 4B). We then asked whether the use of surgical wax might have any inhibitory effect on homing of cancer cells. This was not the case, because injecting MDA cancer cells 1 minute before clot induction did not affect the total number of cancer cells at the site of the clot both in the presence or absence of wax (figure 4C). We next evaluated whether injecting cancer cells at different time points has any effect on homing, and injected cancer cells 15 minutes before, 1 minute before and 5 minutes after clot induction. Here too, there was no difference in the number of cancer cells homing to the bone marrow (figure 4D). We then wondered, whether homing was affected by the length of time since injections. Therefore the number of cancer cells that homed to the clot was evaluated at different time points after clot induction followed by injection of cancer cells. There was no difference in the number of human cancer cells arriving at the site of intervention compared to the opposite side after one, four, 24 and 48 hours in a one-way ANOVA (figure 4E in the −1 min group). A similar experiment was performed, whereby cancer cells were injected after clot induction (+5 min group). At 1 hour, less cancer cells were detected in the hematoma, but this effect was lost at later time points (figure 4F). This suggests that the presence of a blood clot does not offer a permissive environment for homing of cancer cells.

### Metastasis development in the presence of hematoma

We next examined whether the formation of a blood clot was associated with an increase in the chance of metastatic lesion formation. Two experimental mice groups were evaluated. In the first group (−1 min), the mice underwent intracardial cancer cell injection followed 1 minute later by the procedure to induce intratibial clot formation. In the second group (+5 min), the mice underwent the procedure to induce intratibial clot formation, followed by injection of cancer cells into the left ventricle five minutes later. Tumor cells injected intracardially circulate for approximately one hour after which time most cells are cleared from the circulation. Twenty-four hours later no bioluminescence signal can be detected (data not shown). The control sham-operated group received only intracardiac cancer cell injections without clot induction in the left tibia.

**Figure 5:**
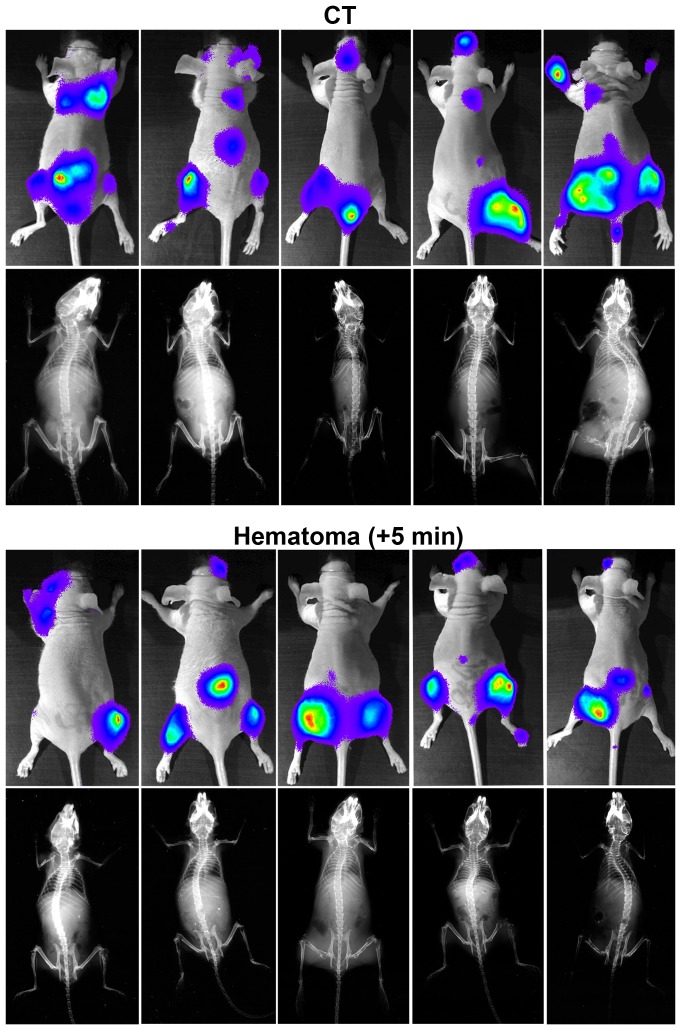
Bioluminescence and x-ray imaging *in vivo*. Representative bioluminescence images from the last measurements before death and x-ray images at the time of death from 5 CT and 5 experimental mice in the group injected with cancer cells 5 minutes after clot induction. Upper panel represents the paired pictures from the CT group and the lower panel represents the paired pictures from the experimental group.

Weekly bioluminescence measurements starting three weeks after cancer cell injection were performed. This was possible because the cancer line used contained a luciferase construct [18]. Since the cancer cell line has been selected to only home to the bone marrow, no lesions outside the skeleton could be detected. Examples from 5 pairs of bioluminescence and x-ray pictures obtained from CT and the +5 min group are presented in figure 5.

**Figure 6:**
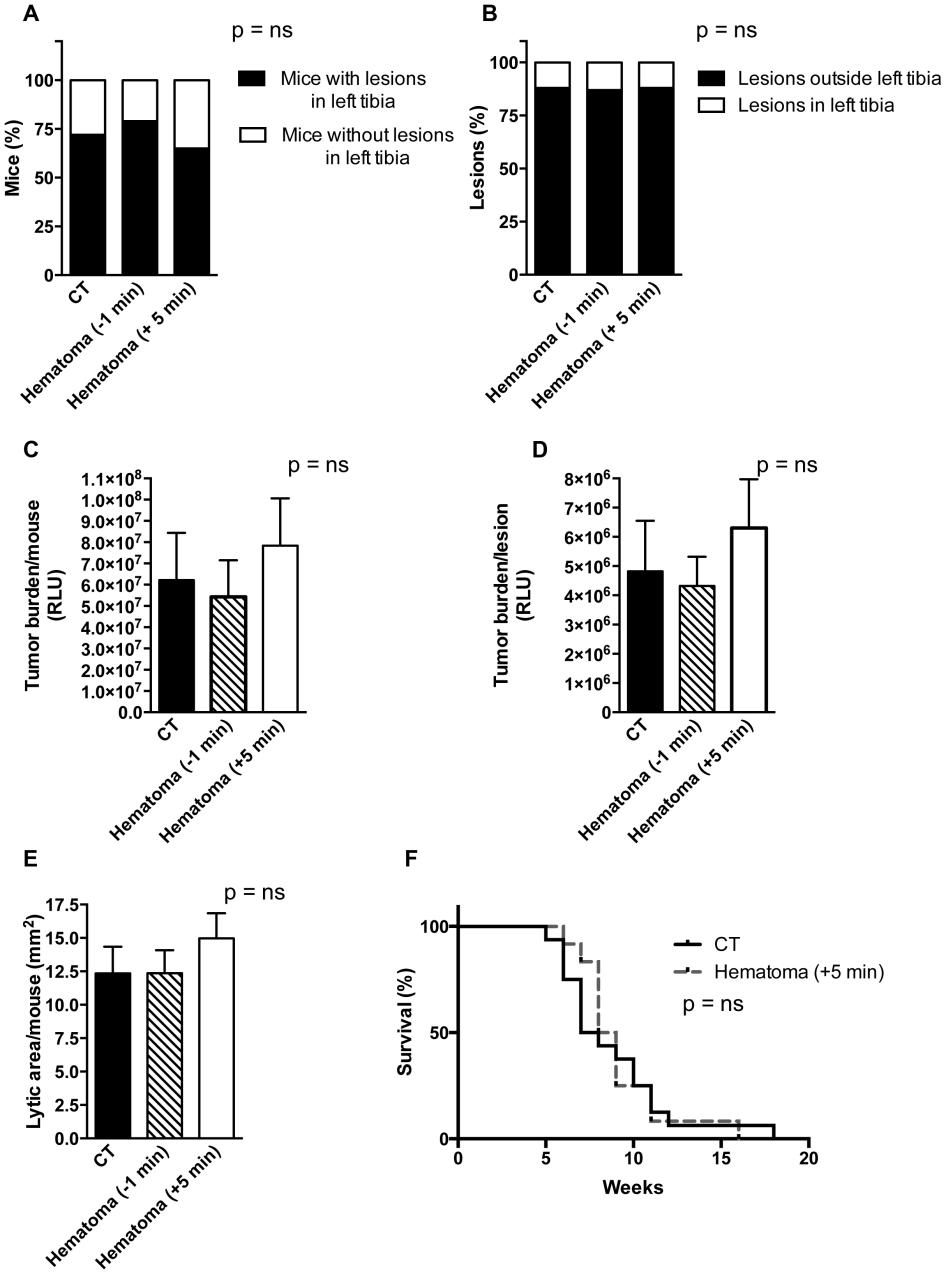
Comparison of control and experimental groups. (A) In the control group 13 lesions developed in the left tibia while in both experimental groups 11 developed in the left tibia. The percentages of mice with lesions in the left tibia is shown. n = 18, 14 and 17 mice. (B) The percentage of left tibia lesions in comparison to the total number of lesions is similar in both CT-experimental group pairs. n as in A. (C) Total bioluminescence signal per mouse expressed in relative light units (RLU) was similar between the control and the experimental groups at 7 weeks. n = 12, 11 and 15 mice. (D) Total bioluminescence signal per lesion was similar too. n as in C. (E) The size of the lytic lesions at 7 weeks was comparable. n as in A. (F) Survival curves of the control group (CT) and the experimental +5 min hematoma group shows that there is no difference in median survival (7.5 vs. 8.5 weeks, p = ns). n as in A. For ease of presentation CT was compared with the group with the seemingly larger median survival only.

Seven weeks after cancer cell injection 13 out of 18 mice (72%) of the mice in the control group developed lesions in the left tibia. Intracardiac cancer cell injection prior to clot induction (−1 min) resulted in 11 affected in the left tibia (out of 14 mice) (79%). Similarly, injection of cancer cells 5 minutes after clot induction resulted in 11 mice affected with left tibia lesions (out of 17 mice) (65%) (figure 6A). Examined differently, a total of 115 lesions had developed in the control group, out of which 13 (11%) were localized in the left tibia and 17 in the right tibia. Intracardiac cancer cell injection prior to intratibial flushing (−1 min) resulted in 85 bone metastatic lesions, out of which 11 (13%) were localized to the left tibia, and 11 in the right tibia (n = 18 and 14 mice, p = ns), while injection of cancer cells 5 minutes after clot induction resulted in 89 bone metastatic lesions, out of which 11 (12%) were localized to the left tibia, and 11 in the right tibia (n = 18 and 17 mice, p = ns) (figure 6B). The summary of the lesion numbers and their locations at 7 weeks after cancer cell injection (including mice that died prior to seven weeks) are presented in Table 1 (sample size was calculated *a priori* for a power of 0.80). This suggests that with a *post hoc* power of 0.89 (−1 min group) and 0.92 (+5 min group) the presence of a blood clot does not affect the development of a macroscopic lesion. Tumor burden was not affected since the total bioluminescence signal per mouse (figure 6C), and per lesion (figure 6D) did not differ between the experimental and the control group. In support of these findings, analysis of x-ray films obtained at seven weeks after cancer cell injection revealed comparable sizes of lytic lesions between the groups (figure 6E). In line with these findings, median survival did not differ between all three groups (CT 7.5 weeks; −1 min group 8.0 weeks; +5min group 8.5 weeks; p = ns for the CT and experimental pairs) (figure 6F).

**Table 1:** Comparison of control and experimental groups.

Number of lesions and their locations (confirmed by bioluminescence and x-ray)	Control group n = 18 (%)*	Hematoma group -1 min n = 14 (%)*	Hematoma group +5 min n = 17 (%)*
Total number of bone lesions	115	85	89
Left tibia (Control or hematoma)	13 (11)	11 (13)	11 (12)
Right tibia	17 (15)	11 (13)	11 (12)
Cranium and jaw	17 (16)	12 (14)	17 (19)
Spine	16 (14)	12 (14)	14 (16)
Ribs	1 (1)	3 (4)	3 (3)
Shoulder	6 (5)	8 (9)	5 (5)
Hip	6 (5)	7 (8)	2 (2)
Femur	7 (6)	4 (5)	8 (9)
Humerus	15 (13)	5 (6)	8 (9)
Forelimb foot	9 (8)	6 (7)	5 (5)
Hindlimb foot	8 (7)	6 (7)	8 (9)
**Number of lesions/mouse**	**6.4**	**6.1**	**5.3**

The number of macroscopic lesions detectable by bioluminescence imaging in the left tibia was similar between control animals that did not undergo clot induction and experimental animals with a blood clot in which cancer cells were injected 1 minute prior to (−1 min), or 5 minutes after clot induction (+5 min). Data from all mice (including those that already died) 7 weeks after cancer cell injection are shown. Data were analyzed using Fisher's exact test and no significant differences were detected in the CT/experimental group pairs.

*The percentage presented is calculated as follows: number of lesions at a specific site/total number of lesions in the group.

Based on these findings, we conclude that the presence of a blood clot does not affect the development of a macroscopic lesion or the growth characteristics of bone metastases in a murine model of breast cancer metastasis.

## Discussion

The principal finding of our study is that the formation of a clot and hematoma in the bone marrow neither increases the chance that a cancer cell becomes incorporated in the clot, nor is it associated with an increase in the chance that a metastatic lesion develops in the area of the clot, nor does it affect the later growth of tumors.

With experimental evidence emerging in 1990s the seed and soil theory that implies that homing and growth of cancer cells is facilitated in locations that provide the right microenvironment, has gained much ground among scientists working on metastasis formation [29]. The accumulation of blood outside a blood vessel results in the activation of the coagulation cascade associated with the formation of a blood clot, with the ensuing accumulation of thrombin and various other proteins that support tumor establishment and growth [9]–[11]. Clot formation is also associated with activation of platelet aggregation, which release SDF-1, VEGF (Vascular endothelial growth factor) and PDGF (platelet-derived growth factor) [14], [30], [31]. These three molecules were shown to exert pro cancerous effects. While SDF-1 normally serves as a chemotaxis signal for platelets and inflammatory cells to initiate the wound healing process [14], [32], it is similarly chemotactic for cancer cells and was shown to stimulate cancer cell migration and establishment in the bone marrow [33]. Therefore, in its presence, cancer cells in the circulation would be expected to proceed to the site of clot formation. Both PDGF and VEGF support angiogenesis [34]–[36]. However angiogenesis is only required at a later time point when the lesion is already about 1 mm^3^ in size [37] indicating that this effect should influence only tumor growth and development but not the homing of circulating cancer cells. Lastly, platelets promote cancer development by impairing the function of natural killer cells [38]. Thus the accumulation of platelets in a blood clot should be permissive to cancer cell homing and may promote growth.

Associated with the flushing of the bone marrow, shear forces on the blood vessels and the sinusoids result in leakage of blood with all its components into the bone marrow cavity. Nevertheless no increase in the number of cancer cells trapped in the clot could be detected. It thus seems that the prometastatic roles of various members of the coagulation cascade and the platelets in the cytokine rich environment of the bone marrow are not critical at physiologic concentrations for the early development of cancer lesions or for their later growth. The lack of effect on growth starting after 3 weeks could however be due to recovery and resolution of the blood clot by then. Lastly, an inhibitory role due to the presence of megakaryocytes cannot be excluded, because megakaryocytes in the bone marrow have been shown to induce apoptosis and decrease proliferation of prostate cancer cells [39].

Bone marrow disruption is associated with the formation of a mineralizing trabecular network and an increase in osteoblasts [40]. Untreated osteoblasts *in vitro* and osteoblasts lining the bone marrow *in vivo* release a variety of cancer promoting cytokines such as interleukin-6 (IL-6) and monocyte chemotactic protein-1 (MCP-1) [4], [41], both of which have also been shown to promote cancer cell migration and invasion [4], [42], [43]. We therefore performed histomorphometric analyses of the bone after clot induction [41]. However, flushing the bone marrow in our model did not induce significant changes in bone formation as shown in figures 2 and 3. This contrasts to histologic changes shown with disruption of the bone marrow and reported before [40], and is in line with the lack of a difference in cancer development between the flushed tibia and the opposite sides. The clot induction was not associated with an increase in bone resorption either. Therefore, the release of growth factors from the bone matrix in our model of clot induction is limited [44]. Thus, our model stands in contrast to fracture models where the broken bone results in the formation of a blood clot followed by infiltration by inflammatory cells, formation of the fibrocartilagenous callus, and finally bone remodeling resulting in normal bone structure in the area of the fracture. Even though inflammation is associated with the release of some cytokines that affect bone cells, and hence a low level of bone remodeling with release of growth factors from the matrix might ensue during the early stages after fracture formation [45]–[47], the major remodeling step during fracture healing takes place after the blood clot had resolved [48]. Our experimental clot induction model examines the effect of blood clot formation within the cytokine-rich environment of the bone marrow. Because of the absence of measurable bone remodeling it thus does not include the role of the growth factors released from the matrix during remodeling. However, the bone marrow itself is a microenvironment already rich with cytokines that can promote cancer cell homing and growth [49], whereby the same cytokines required for hematopoiesis seem to be beneficial for homing of cancer cells to the bone marrow and tumor growth. One such cytokine is SDF-1 that is involved in hematopoiesis and chemotaxis [14], [50]. Furthermore, osteoblasts release a variety of cancer promoting cytokines that support cancer development as discussed [4], [42], [43]. The similar homing and cancer growth between the flushed side and the opposite side in our model therefore suggests that blood clots even in a cancer-promoting environment do not necessarily support cancer formation.

The decision to introduce cancer cells by intracardiac injection was based on the need to provide for a large number of circulating cancer cells looking for a home during clot induction [51]. This model thus seems better suited to examine the role of clot proteins in supporting cancer cell homing and growth than introducing a cancer, from which the cells would first need to move out and roam in the blood stream before migrating into the clot [27]. By using two different time points for the injection of cancer cells in relationship to induction of blood clotting we evaluated both the role of the initial thrombin surge and acute release of clotting factors on already circulating cancer cells (in the −1 min model) and the role of the presence of a clot prior to the surge in circulating cancer cells (in the +5 min model). In the case of introduction of the cancer cells 5 minutes after clot induction (+5 min) the decrease in the number of cancer cells that homed to the bone marrow one hour after clot induction was surprising. In particular since we had expected an increase in homing of cancer cells to the clot driven by the availability of the various clotting factors. This decrease could be explained by the volume taken up by the blood clot that results in a decrease in the volume of circulating blood in the bone marrow and hence a decrease in the number of cancer cells [51]. A related possible explanation is that the blood clot does not allow infiltration by cancer cells due to the accumulation of matrix. This early effect however does not bear any relevance to the number of cancer cells at the site of clot after 24 hours or affect the development of cancerous lesions. While our data do not allow for a conclusion with regard to whether a larger clot might have caused an increase in the homing of cancer cells, it seems reasonable to conclude, based on our findings, that the formation of a 0.5 mm^2^ clot (as shown in figure 1E) is not associated with increased homing of cancer cells to the clot after 24 hours.

Even though this experimental model compares favorably to other cancer models because it is based on a human cancer cell line and shares the ability to induce bone metastatic lesions with the clinical human counterpart, other cancer types might be more responsive to various cancer-promoting effects of physiologic concentrations of blood clot components. Nevertheless, it seems safe to predict that the effect in other models is probably limited in particular since two further cell lines failed to show an increase in homing of cancer cells to the blood clot.

## Conclusions

Based on our findings we therefore conclude that, in a mouse model of human breast cancer, induction of a hematoma/clot did not promote bone metastasis formation or growth. Accordingly, there is currently no experimental evidence to support the possibility of metastasis formation in freshly injured areas in patients with cancer. It seems more likely that the coincidence of metastasis at sites of surgical interventions or trauma is due exclusively to chance.
