# Correction: Blood Clot Formation Does Not Affect Metastasis Formation or Tumor Growth in a Murine Model of Breast Cancer

**DOI:** 10.1371/journal.pone.0103911

**Published:** 2014-07-23

**Authors:** The PLOS ONE Staff

The fourth author's name is spelled incorrectly. The correct name is: Marco G. Cecchini. The correct citation is: Rossnagl S, von Au A, Vasel M, Cecchini MG, Nakchbandi IA (2014) Blood Clot Formation Does Not Affect Metastasis Formation or Tumor Growth in a Murine Model of Breast Cancer. PLoS ONE 9(4): e94922. doi:10.1371/journal.pone.0094922
